# Ionizing Radiation and Estrogen Affecting Growth Factor Genes in an Experimental Breast Cancer Model

**DOI:** 10.3390/ijms232214284

**Published:** 2022-11-18

**Authors:** Gloria M. Calaf, Leodan A. Crispin, Juan P. Muñoz, Francisco Aguayo, Debasish Roy, Gopeshwar Narayan

**Affiliations:** 1 Instituto de Alta Investigación, Universidad de Tarapacá, Arica 1000000, Chile; 2 Laboratorio de Oncovirología, Programa de Virología, Instituto de Ciencias Biomédicas (ICBM), Facultad de Medicina, Universidad de Chile, Santiago 8380000, Chile; 3 Department of Natural Sciences, Hostos College of the City University of New York, New York, NY 10451, USA; 4 Department of Molecular and Human Genetics, Banaras Hindu University, Varanasi 221005, Uttar Pradesh, India

**Keywords:** radiation, breast cancer, estrogen, *FGF2*, *FGFBP1*, *TGFA*, *TGFBR3*, *IGF1R*

## Abstract

Genes associated with growth factors were previously analyzed in a radiation- and estrogen-induced experimental breast cancer model. Such in vitro experimental breast cancer model was developed by exposure of the immortalized human breast epithelial cell line, MCF-10F, to low doses of high linear energy transfer (LET) α particle radiation (150 keV/μm) and subsequent growth in the presence or absence of 17β-estradiol. The MCF-10F cell line was analyzed in different stages of transformation after being irradiated with either a single 60 cGy dose or 60/60 cGy doses of alpha particles. In the present report, the profiling of differentially expressed genes associated with growth factors was analyzed in their relationship with clinical parameters. Thus, the results indicated that Fibroblast growth factor2 gene expression levels were higher in cells transformed by radiation or in the presence of ionizing radiation; whereas the fibroblast growth factor-binding protein 1gene expression was higher in the tumor cell line derived from this model. Such expressions were coincident with higher values in normal than malignant tissues and with estrogen receptor (ER) negative samples for both gene types. The results also showed that transforming growth factor alpha gene expression was higher in the tumor cell line than the tumorigenic A5 and the transformed A3 cell line, whereas the transforming growth factor beta receptor 3 gene expression was higher in A3 and A5 than in Tumor2 cell lines and the untreated controls and the E cell lines. Such gene expression was accompanied by results indicating negative and positive receptors for transforming growth factor alpha and the transforming growth factor beta receptor 3, respectively. Such expressions were low in malignant tissues when compared with benign ones. Furthermore, Fibroblast growth factor2, the fibroblast growth factor-binding protein 1, transforming growth factor alpha, the transforming growth factor beta receptor 3, and the insulin growth factor receptor gene expressions were found to be present in all BRCA patients that are BRCA-Basal, BRCA-LumA, and BRCA-LumB, except in BRCA-Her2 patients. The results also indicated that the insulin growth factor receptor gene expression was higher in the tumor cell line Tumor2 than in Alpha3 cells transformed by ionizing radiation only; then, the insulin growth factor receptor was higher in the A5 than E cell line. The insulin growth factor receptor gene expression was higher in breast cancer than in normal tissues in breast cancer patients. Furthermore, Fibroblast growth factor2, the fibroblast growth factor-binding protein 1, transforming growth factor alpha, the transforming growth factor beta receptor 3, and the insulin growth factor receptor gene expression levels were in stages 3 and 4 of breast cancer patients. It can be concluded that, by using gene technology and molecular information, it is possible to improve therapy and reduce the side effects of therapeutic radiation use. Knowing the different genes involved in breast cancer will make possible the improvement of clinical chemotherapy.

## 1. Introduction

Breast cancer is a complex disease in which multiple genetic alterations are present. However, its origin is not known, and it is generally associated with the accumulation of multiple anomalies in individual cells related to specific sequences of genetic changes that are important in tumor progression [1,2]. Cancer originates through numerous steps that lead to transformed cells accumulating genetic changes that codify regulatory proteins.

The risk of breast cancer is associated with endogenous factors such as hormones and exogenous factors such as radiation exposure (atomic bomb) or chemical factors from the environment. It is known that the transformation of a normal cell to a malignant one is induced by mutations in the genes that codify proteins, important in the regulation of cell processes that contribute to carcinogenesis through complex biochemical pathways [3]. There exists a high probability that radiation and estrogen can be one of the causes of breast cancer [4,5].

Physical carcinogens such as radiation have been used as initiators and promoters in cell models of human and animal cell lines [6,7,8]. In particular, it has been demonstrated in fractionated radiation of x-rays in human breast epithelial cells [9]. Radon is a noble gas, radioactive emanated from the disintegration of radium. It originates in alpha particles, a highly energetic ionizing substance that, when it is inhaled, is converted into substances that cause several pathologies. Its levels are accumulated inside old houses [10,11].

The identification of genes involved in cancer stages in estrogen and radiation-induced breast carcinogenesis is of critical importance for a better understanding of the mechanisms involved. An experimental breast cancer model has been established where a normal human breast epithelial cell line such as MCF-10F underwent a stepwise transformation into malignant cells by the effect of low doses of alpha-particles in the presence of estrogens [6].

The fibroblast growth factor-binding protein 1 (*FGFBP1*) induces the FGF receptor (FGFR). Fibroblast growth factor2 (*FGF2*)-mediated angiogenesis is hypothesized to play a part in the development of squamous cell, colon, and breast cancers [12,13].

Transforming Growth Factor Alpha (*TGFA*) and Transforming Growth Factor beta (*TGFB*) are secreted by a wide range of altered cells and malignancies [14,15,16], and then, it is assumed that is an autocrine growth factor in the development and maintenance of cancer. TGF superfamily signaling is modulated by the type III transforming growth factor (*TGF*) receptor [17]. Transforming Growth Factor beta (TGF)-beta type III receptor (*TGFBR3*) is a membrane proteoglycan that often functions as a coreceptor with other TGF-beta receptor superfamily members.

The insulin growth factor receptor (*IGF1R*) is a tyrosine kinase receptor that has been linked to the genesis and progression of breast cancer tumors, and its *IGF1R* expression is associated with clinical–pathological factors in breast carcinomas [18]. The present study aimed to analyze the differential expression of genes related to growth factors such as *FGF2*, *FGFBP1*, *TGFA*, *TGFBR3*, and *IGF1R* in a radiation- and estrogen-induced experimental breast cancer model.

## 2. Results

### 2.1. An Experimental Radiation and Estrogen-Induced Breast Cancer Model

An in vitro experimental breast cancer model was developed by exposure of the immortalized human breast epithelial cell line, MCF-10F, to low doses of high linear energy transfer (LET) α particle radiation and subsequent growth in the presence or absence of 17β-estradiol. The MCF-10F cell line was analyzed in different stages of transformation after being irradiated with either a single 60 cGy dose or 60/60 cGy doses of alpha particles that then showed gradual phenotypic changes, including altered morphology, an increase in cell proliferation relative to the control, anchorage-independent growth and invasive capability, before becoming tumorigenic in nude mice. The cell lines used in this model were: (i) the parental cell line MCF-10F (C), (ii) an Estrogen cell line (E), (iii) a malignant and nontumorigenic cell line (60/60 cGy) named Alpha3 (A3), (iv) a malignant and tumorigenic cell line (60/60 cGy plus estrogen) named Alpha5 (A5), and (v) the Tumor2 cell line (T2) derived from a xenograft of the A5 cell line injected into nude mice; these cell lines were cultured in the presence or absence of E for periods of up to 10 months post-irradiation [6].

### 2.2. Breast Cancer Model Induced by Radiation and Estrogen: Gene Expression Determined by Profiling of Differentially Expressed Genes through an Affymetrix Array (U133A)

**Figure 1:**
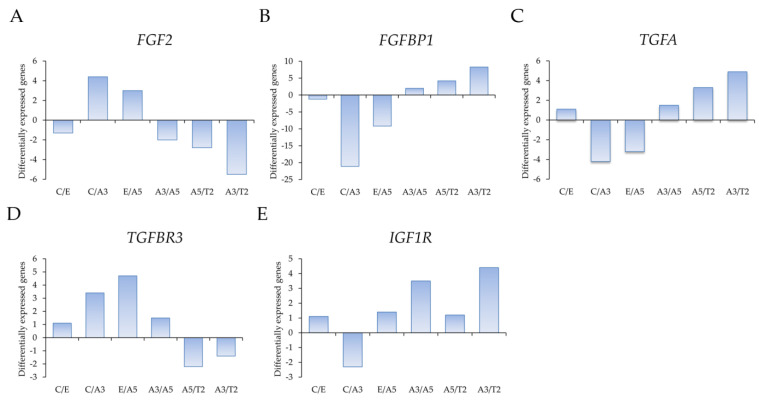
(**A**) Profiling of differentially expressed genes obtained through an Affymetrix array (U133A) data comparing (**A**) *FGF2*, (**B**) *FGFBP1*, (**C**) *TGFA*, (**D**) *TGFBR3*, and (**E**) *IGF1R* in the following cell lines: MCF-10F/Estrogen (C/E), Control/Alpha3 (C/A3), Estrogen/Alpha5 (E/A5), Alpha3/Alpha5 (A3/A5), Alpha5/Tumor2 (A5/T2), and Alpha3/Tumor2 (A3/T2). The graph was obtained from a cluster-dendrogram repository of gene expression from our laboratory for this article.

Growth factor gene expression was determined by the profiling of differentially expressed genes through an Affymetrix array (U133A) (Figure 1), such as (A) *FGF2*, (B) *FGFBP1*, (C) *TGFA*, (D) *TGFBR3*, and (E) *IGF1R* in the following cell lines: MCF-10F/Estrogen (C/E), Control/Alpha3 (C/A3), Estrogen/Alpha5 (E/A5), Alpha3/Alpha5 (A3/A5), Alpha5/Tumor2 (A5/T2), and Alpha3/Tumor2 (A3/T2). 

The results indicated that high LET radiation such as that emitted by radon progeny in the presence of estrogen induced a cascade of events indicative of cell transformation and tumorigenicity in human breast epithelial cells. The studies are associated with the genes involved in the control of changes induced by growth factors that take place by the effect of radiation and hormones in the normal nontumorigenic MCF-10F and the tumorigenic cell lines.

When a comparison was made between genes affected in the model as *FGF2* and FGFBP1 gene expression, the results indicated that both had opposite results. Thus, while *FGF2* was higher in A3 (60cGy/60cGy) than in the T2 cell lines, *FGFBP1* was higher in T2 than in the A3 cell lines. Such results indicated that radiation (60cGy/60cGy doses) increased FGF2, whereas, in T2, 60cGy/60cGy doses plus estrogen injected in the immunologically depressed nude mouse. *FGFBP1* was higher than the A3 cell line, indicating that the gene is at a higher level in the tumor and is influenced by estrogens, being a good marker for breast carcinogenesis. *FGF2* was also higher in the A3 cell line than in the control MCF-10F (C) cell line. It is important to mention that, when single doses of alpha radiation (60cGy) were used to develop this model, there were no signs of transformation; then, the double (60cGy/60cGy) doses induced signs of transformation such as invasive capabilities and anchorage independence, but it was not tumorigenic in the SCID or nude mice [6]. FGF3 was even higher in the A5 (60cGy/60cGy doses plus estrogen) cell line when compared with the E cell line, confirming the estrogen effect in such a process. However, *FGFBP1* was higher in the C than A3 cell line and in E than the A5 cell line.

When a comparison was made between *TGFA* and *TGFBR3* gene expression, studies indicated opposite results. Thus, the TGF alpha gene expression was higher in T2, the tumor cell line, than the nontumorigenic A3 cell line, as well as the tumorigenic cell line A5, indicating radiation in the first case, and the environment of the tumor formed in the second case added other factors that it could also be growth factors present in the animal. However, *TGFBR3* gene expression was higher in the A5 than E cell lines and in the A3 than C cell lines, indicating the presence of radiation in the role of such a gene. It was also important to see that *IGF1R* gene expression was higher in the tumor cell line T2 than the nontumorigenic A3 cell line and this one compared to the control cell line.

### 2.3. Differential Gene Expression Levels between Tumor and Normal Tissues across Various Cancer Types

**Figure 2:**
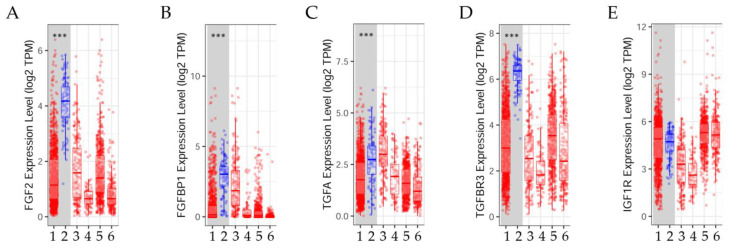
Gene expression levels of (**A**) fibroblast growth factor 2 (*FGF2*), (**B**) the fibroblast growth factor binding protein 1 (*FGFBP1*), (**C**) transforming growth factor alpha (*TGFA*), (**D**) transforming growth factor beta receptor 3 (*TGFBR3*), and (**E**) insulin-like growth factor 1 receptor (*IGF1R*) in different human cancers compared with adjacent normal tissue in the Tumor Immune Estimation Resource (TIMER2.0) database [19]. (1) BRCA.Tumor (*n* = 1093), (2) BRCA.Normal (*n* = 112), (3) BRCA-Basal.Tumor (*n* = 190), (4) BRCA-Her2.Tumor (*n* = 82), (5) BRCA-LumA.Tumor (*n* = 564), (6) BRCA-LumB.Tumor (*n* = 217).

Data on the *FGF2*, *FGFBP1*, *TGFA*, *TGFBR3*, and *IGF1R* expression levels (Figure 2A–E) were analyzed using TIMER 2.0 that matched TCGA tumor tissue with normal tissue [19]. Distributions of gene expression levels are displayed using box plots. The statistical significance computed by the Wilcoxon test is annotated by the number of asterisk (*: *p*-value < 0.05, **: *p*-value < 0.01, and ***: *p*-value < 0.001).

The results indicated that the *FGF2*, *FGFBP1*, *TGFA*, and *TGFBR3* gene expression levels were significantly (*p* < 0.001) lower in malignant breast cancer than in normal tissues; however, the *IGF1R* expression levels were not significantly different between both types of tissues (Figure 2A–E).

### 2.4. Gene Expression and Estrogen Receptor Status in TCGA Breast Cancer

**Figure 3:**
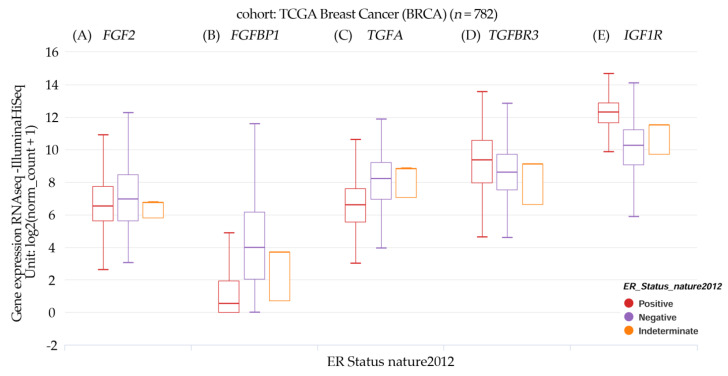
Box plot transcript expression of (**A**) *FGF2*, (**B**) *FGFBP1*, (**C**) *TGFA*, (**D**) *TGFBR3*, and (**E**) *IGF1R* in breast cancer (TCGA, *n* = 782) stratified by nature2012 for the estrogen receptor status (One-way ANOVA, *p* < 0.05). Raw data were extracted from the University of California, Santa Cruz (http://xena.ucsc.edu/). UCSC Xena functional genomics explorer (accessed 21 August 2021, https://xenabrowser.net) [20].

The results in Figure 3A–E from the UCSC Xena online exploration tool [20] indicated that patients’ samples with high FGF2, *FGF2*, *FGFBP1,* and *TGFA* gene expression had negative ER expression, and those with high *TGFBR3* and *IGF1R* gene expression were positive for ER. 

### 2.5. Correlation between EGFR and Growth Factor Genes in Breast Invasive Carcinoma Subtypes

TIMER2.0 was used to identify the correlation between *EGFR* and other gene expressions in breast-invasive carcinoma subtypes with purity adjustments such as *FGF2*, *FGFBP1*, *TGFA*, *TGFBR3*, and *IGF1R* in all BRCA patients.

**Figure 4:**
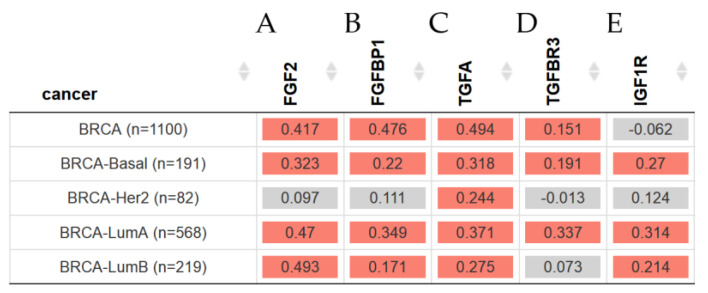
The heatmap table shows the correlation between *EGFR* expression and (**A**) *FGF2*, (**B**) *FGFBP1,* (**C**) *TGFA*, (**D**) *TGFBR3*, and (**E**) *IGF1R* gene expression level in breast invasive carcinoma subtypes with purity adjustment. The red color indicates a statistically significant positive correlation (Spearman’s, *p* < 0.05), and gray denotes a nonsignificant result. The expression level was estimated by TIMER2.0 [19].

The results in Figure 4A–E show the Heatmap table, indicating that *EGFR* was significantly (*p* < 0.05) correlated with *FGF2*, *FGFBP1*, *TGFA*, and *TGFBR3* in all BRCA patients, except for the *IGF1R* expression levels in Her2. The results showed a significantly (*p* < 0.05) positively correlation with all these genes in BRCA-Basal and BRCA-LumA and BRCA LumB, except for *TGFBR3,* which was negative for BRCA-Her2 and BRCA-LumB and positive for BRCA LumB.

Among all BRCA patients, TGFBR3 was positive in all BRCA patients and other types such as BRCA-Basal and BRCA-LumA. The correlation between EGFR expression and *FGF2* and *FGFBP1* gene expression levels in breast invasive carcinoma subtypes with purity adjustment was statistically significant in its positive correlation in all BRCA, except for BRCA-Her2. Such results showed that EGFR was significantly (*p* < 0.05) correlated with *FGF2* and *FGFBP1* gene expression levels in all BRCA patients and significantly (*p* < 0.05) positively correlated with all these genes in BRCA patients. Thus, such expression was higher in BRCA-Basal, BRCA-HER, BRCA-LumA, and BRCA–LumB patients. 

### 2.6. Gene Expression and Clinical-Stage Factors across Various Breast Cancer Subtypes

The association between the gene expression and the stage of the disease is shown in Table 1. The clinical relevance of *FGF2*, *FGFBP1*, *TGFA*, *TGFBR3*, and *IGF1R* expression was explored using the TIMER2.0 Gene Outcome Module adjusted by the clinical stage factors across various cancer types. 

The results in Table 1 indicate that the *FGF2*, *FGFBP1*, *TGFA*, *TGFBR3*, and *IGF1R* gene expression levels were significantly (*p* < 0.001) higher in all BRCA in stages 3 and 4 than in other clinical stages of patients. Such levels were non-significantly different in any BRCA-Basal patients. In addition, elevated *FGF2* and *TGFA* gene expression levels were significant (*p* < 0.001) in stage 4 in BRCA-LumA in comparison with other clinical stages. There was a significant difference in stage 4 of patients in BRCA-LumB (*p* < 0.01) and in BRCA-Her2 (*p* < 0.05). The *FGFBP1* gene expression levels were also significantly (*p* < 0.05) higher in stage 4 in BRCA-Her2 and BRCA-LumB patients, and it was significant (*p* < 0.01) in stage 4 in BRCA-LumA patients. The *TGFBR3* gene expression levels were significantly (*p* < 0.001) higher in stage 4 in BRCA-LumA patients, and there was also a significant (*p* < 0.01) difference in stage 4 in BRCA-Her2 and BRCA-LumB patients. The *IGF1R* gene expression levels were also significantly (*p* < 0.001) higher in stage 4 in BRCA-LumA patients and there was a significant (*p* < 0.05) difference in stage 4 in BRCA-Her2 and BRCA-LumB patients. When the clinical stage was compared with BRCA-Basal, they were all negative; The *IGF1R* gene expression was significantly higher in Stages 3 and 4 than in the other clinical stages of patients. The *IGF1R* gene expression levels were also significantly (*p* < 0.001) higher in stage 4 in BRCA-LumA patients, and there was a significant (*p* < 0.05) difference in stage 4 in BRCA-Her2 and BRCA-LumB patients; however, this expression was not significant in BRCA-Basal patients, corroborating the difficulties of designing drugs for such patients, and there was a correlation between *EGFR* and *IGF1R* gene expression. Among all BRCA patients, IGF1R was negative in all BRCA patients but positive in BRCA-Basal, BRCA-LumA, and BRCA-LumB.

## 3. Discussion

The present report shows growth factor genes linked to human breast cancer through an experimental breast cancer model that derived from a normal breast cell line transformed into malignant ones. These studies indicated that high LET radiation such as that emitted by radon progeny, in the presence of estrogen, induced a cascade of events indicative of cell transformation and tumorigenicity in human breast epithelial cells. 

Overexpression of several oncoproteins and tumor suppressor genes was reported where growth factors were thought to be involved in such processes [6,21]. Gene expression microarrays have been a useful tool for comparing and contrasting cell lines and disease states [22,23]. Biological annotations such as the Gene Ontology (GO) or the Kyoto Encyclopedia of Genes and Genomes (KEGG) pathways have been used in studies to extrapolate biological roles and regulatory associations from changes in individual genes [24]. As a result, various microarray studies have identified similarities and differences in mRNA expression levels among samples [25,26,27,28,29]. Single genes or groups of related genes are typically associated with many biological activities, and these activities usually correspond to changes in gene expression across distinct tissue types [30].

To find novel prognostic/diagnostic markers for different forms of cancer, in vitro model systems have been widely employed to obtain insights into the molecular events of cancer, such as initiation, promotion, and prevention. All of these elements have biological consequences that either result in cellular death or cell survival, such as various mutations [31] that have an impact on different genes and either activate proto-oncogenes or inactivate tumor suppressors [32,33]. In this situation, the expression of tumor suppressor genes (that restrict cell proliferation), oncogenes (that regulate cancer cell migration and proliferation), and checkpoint genes, which regulate DNA repair and control cell cycle, can be altered. Gene expression changes that occur when cells alter their phenotype lead to cell proliferation, which, in turn, causes a loss of cell–cell adhesion, migration, and invasion into other tissues or organs through blood vessels, such as the epithelial-to-mesenchymal transition (EMT) [34].

The soil and porous rocks that contain uranium give off the radioactive gas known as radon. All people are exposed to radon to some extent, although it is more prevalent in regions with high uranium concentrations, because most ground sources contain trace amounts of the element. Radon builds up in poorly ventilated or enclosed spaces when it migrates from soil and rock into the surrounding air. These regions serve as the main settings in which people are exposed to radiation from radon and suffer negative health effects. Since it is so common, residential radon is crucial. Even though miners may be exposed to much higher levels of ionizing radiation, even a small amount of extra risk from radon exposures in a residential setting has the potential to cause far more cancer cases in the general population than the exposure of a relatively small number of miners [35].

Ionizing radiation consists of ions, as well as positively and negatively charged particles, such as alpha particles and electrons [31]. The radioactive decay of heavy elements like radium, plutonium, or thorium that are released into the body from a radioactive source damages the cells and, as a result, the DNA in the body; alpha particles are formed by two neutrons and two protons from the atom’s nucleus during the decrease of the atomic mass number and reduction of the atomic number [36]. Since it may quickly penetrate tissues to reach a particular spot, ionizing radiation is a good substitute for conventional cancer therapy [37]. Irradiated radioisotopes are used for both diagnosis and treatment [38]. Breast tumors are treated with radiotherapy frequently [39,40]. Direct ionization or water radiolysis by ionizing radiation can alter and destroy DNA, RNA, and cell membrane components such as lipids and proteins [41]. Numerous reactive oxygen species (ROS) are produced during water radiolysis, which is known to be the primary mechanism causing tissue damage and cell death [42,43]. Due to increased cellular activity, radiation can also cause inflammation that is linked to tumor progression, such as breast carcinogenesis, which is induced by several factors connected to hormonal development [44,45].

Reports have indicated that early defects in proliferation increase cancer risk, as it occurs in hyperplasia, since it perturbs the pathways usually regulated by local growth factors and systemic hormones, such as estrogens [6]. The estrogen dependence of breast cancer has long been recognized; however, the role of 17beta-estradiol in cancer initiation was not known until some authors showed that it induces complete neoplastic transformation of the human breast epithelial cells MCF-10F [46,47]. They demonstrated that estrogens induced high colony efficiency, loss of ductulogenesis, and invasiveness in Matrigel invasion chambers in MCF cells. The functional profiling of dysregulated genes revealed progressive changes in the integrin signaling pathway, inhibition of apoptosis, acquisition of tumorigenic cell surface markers, and EMT. In tumorigenic MCF-10F cells, E-cadherin was low, whereas HRAS and transforming growth factor beta1 (TGFbeta1) were high. The phenotypic and genomic changes triggered by estrogen exposure that lead normal cells to tumorigenesis confirm the role of this steroid hormone in cancer initiation. There are two hypotheses on the causes of tumor formation, according to Yager and Davidson (2006). The first is that estrogens promote cell proliferation by increasing cell divisions, which increases the likelihood of mutational errors. They suggest that, when enough mutations are left uncorrected, breast cancer can occur. According to a second notion, estrogens can be converted into genotoxic byproducts that produce reactive oxygen species and result in mutations [48].

Our experimental breast cancer model showed that transformed MCF-10F cell lines had a very complex pattern of gene and protein expressions after being exposed to double doses of alpha particles and then treated with estrogen as compared to a single dose of radiation and control; during the transformation process, several changes were induced, such as anchorage independency and invasive capabilities by single or double doses of radiation, either in the presence or absence of estrogen [6].

The association between breast cancer development and prolonged exposure to estrogen suggests that this hormone may have an etiologic role in the causation of this disease [14,15,49]. Estrogen causes breast cancer by inducing proliferation through the estrogen receptor alpha (ER-alpha) [50]. ER-alpha is one of the two recognized ER subtypes, and a great amount of study has been done since the cloning of the ER-alpha. Recent evidence revealed that, at every level of signal transduction, the ER and growth factor-signaling pathways crossed and directly interacted [51], indicating ER–growth factor synergism documented in both normal breast development and, more crucially, in breast cancer progression and antiestrogen resistance. Breast cancer progresses from a state of normally regulated cell proliferation to one of severely aberrant dysregulation and that advancement is linked to an increase in genetic/genomic instability in the animal [21,52,53,54].

Four receptor tyrosine kinases, known as *EGFR (HER1)*, *HER2*, *HER3*, and *HER4*, are part of the human epidermal growth factor receptor (*EGFR/ErbB*) family and are crucial in controlling cell proliferation, differentiation, and migration [55,56]. These studies identified the correlation between *EGFR* and *FGF2*, *FGFBP1*, *TGFA*, TGFBR3, and *IGF1R* in breast-invasive carcinoma subtypes. Thus, *EGFR* was correlated with *FGF2*, *FGFBP1*, *TGFA*, and *TGFBR3* in all BRCA patients, except for the *IGF1R* expression levels in BRCA-Her2. The results also showed a positive correlation with all these genes in BRCA-Basal and BRCA-LumA and BRCA LumB, except for *TGFBR3*, which was positive in BRCA-Her2 and negative for BRCA-Her2 and BRCA-LumB.

The amplification of different members of the FGF family of genes was shown to be a better independent prognostic indicator of breast cancer in humans [57,58], whereas *FGF2* plays important roles in tissue development and repair. Using heparan sulfates (HS)/heparin as a cofactor, FGF2 binds to *FGFR* and induces downstream signaling pathways, such as the ERK pathway, that regulate cellular behavior. FGF-BP1 is a secreted chaperone that mobilizes paracrine-acting FGFs, stored in the extracellular matrix, and presents them to their cognate receptors. *FGFBP1* enhances *FGF* signaling, including angiogenesis during cancer progression, and is upregulated in various cancers.

The *TGFA* gene encodes a growth factor that is a ligand for the epidermal growth factor receptor, which activates a signaling pathway for cell proliferation, differentiation, and development. This protein may act as either a transmembrane-bound ligand or a soluble ligand. 

The encoded receptor named *TGFBR3* is a membrane proteoglycan that often functions as a co-receptor with other TGF-beta receptor superfamily members and may inhibit *TGFB* signaling. A decreased expression of this receptor has been observed in various cancers. It is known that *TGF* stimulates anchorage-independent cell growth in soft agar in the presence of transforming growth factor β [16,59,60,61]. Since *TGF* binds to *EGFR* and causes tyrosine phosphorylation of the receptor [59], it is plausible to consider that both substances influence cancer signaling pathways.

IGF-1R is a protein found on the surface of human cells that belongs to the large class of tyrosine kinase receptors. It is a transmembrane receptor that is activated by a hormone called insulin-like growth factor 1 (*IGF-1*) and by a related hormone called *IGF-2*. It is important to mention that all gene expressions analyzed in these studies showed that the growth factors were positive in BRCA-Basal patients, an important fact considering the absence of treatment in those patients negative for estrogen receptors.

Furthermore, the results suggest that all these markers should be used in the earlier stages of the disease, since an analysis of BRCA patients indicated that the *FGF2*, *FGFBP1*, *TGFA*, *TGFBR3*, and *IGF1R* gene expression levels were present in late stages 3 and 4 of breast cancer.

## 4. Materials and Methods

### 4.1. Cell Lines

MCF-10F cells, obtained from ATTC, were grown in DMEM/F-12 (1:1) medium supplemented with antibiotics (100 U/mL penicillin, 100 μg/mL streptomycin, 2.5 μg/mL amphotericin B (all from Life Technologies, Grand Island, NY, USA)), and 10 μg/mL and 5% equine serum (Biofluids, Rockville, MD, USA), 0.5 μg/mL hydrocortisone (Sigma, St. Louis, MO, USA), and 0.02 μg/mL epidermal growth factor (Collaborative Research, Bedford, MA, USA) were added [6,62,63,64,65,66,67]. The immortalized human breast epithelial cell line MCF-10F was exposed to low doses of high LET particle radiation (150 keV/m), and subsequent growth in the presence or absence of 17-estradiol was utilized to construct an in vitro experimental breast cancer model (Alpha model). Human breast epithelial cells in various phases of transformation made up this model: (i) a control cell line (MCF-10F), (ii) MCF-l0F continually treated with estradiol at 10–8 M (E or Estrogen) (Sigma-Aldrich) named Estrogen cell line, (iii) a malignant cell line (Alpha3), (iv) a malignant and tumorigenic cell line (Alpha5), and (v) the Tumor2 cell line derived from cells originating from a tumor after injection of Alpha5 cells in nude mice [6].

### 4.2. Affymetrix HG-U133A Plus 2.0 GeneChip Microarray Gene Expression Analysis

The breast cancer model (Alpha-model) consists of: (i) MCF-10F, (ii) Estrogen, (iii) Alpha3, (iv) Alpha5, and (v) Tumor2 cell lines that were used to analyze the gene expression by the Affymetrix U133A oligonucleotide microarray (Affymetrix, Santa Clara, CA, USA), which contains 14,500 genes. The arrays were quantitatively analyzed for gene expression using Affymetrix GeneChip Operating Software (GCOS) with a dual global scaling option in the Genes@Work software platform of the discovery algorithm SPLASH (structural pattern localization analysis by sequential histograms) with a false discovery rate of 0.05 [68].

### 4.3. Gene Expression Analysis and Statistical Analysis

TIMER2.0, an information web resource, evaluates the clinical impact of different immune cells in various cancer types through three components: immune association, cancer exploration, and immune estimation, each with several modules to investigate tumor immunological, clinical, and genomic features. The Gene_DE module of the exploration component provided statistical analysis carried out by the Wilcoxon test used in Figure 2 to identify the genes that were upregulated or downregulated in the tumors compared to normal tissues for each cancer type, and the Gene_Corr module provided the statistical analysis carried out by the Spearman’s test to explore the correlation between the desired gene and a list of genes in various cancer types. After submitting “EGFR” with the list of genes in this module, TIMER2.0 automatically generated their relationship (Figure 4). The Gene_Outcome module with an analysis carried out by a Z-Score test provided the clinical relevance of gene expression across various cancer types by the cell-clicking function of the heatmap table to show the Kaplan–Meier curve and the stages of the disease in various cancer types (Table 1) [19].

UCSC Xena, a web source of information, provided the statistical significance computed by the one-way ANOVA test. The UCSC Xena web source allows users to explore functional genomic data sets for correlations between genomic and/or phenotypic variables and focuses on the integrative visualization of multi-omics data sets across different genomic contexts, including genes, genomic elements, or any genomic region, for both coding and noncoding parts of the genome [20]. *p* < 0.05 was considered significant.

## 5. Conclusions

An experimental human breast cancer model derived from the MCF-10F cell line treated with different doses of high-LET (α-particle) radiation and estrogen exposure was used in these studies. It can be concluded that finding new markers such as *FGF2*, *FGFBP1*, *TGFA*, *TGFBR3*, and *IGF1R* could give a clue on how to define combined drug treatments. It is important to mention that there is no drug therapy for negative estrogen receptor patients; therefore, searching for markers for this type of cancer is essential for breast cancer patients. It can be summarized that, in the breast cancer model, the cell line transformed only by radiation independently of estrogen was characterized by greater growth factor gene expression than in the other cell lines. Understanding the effect of radiotherapy on different types of cells will help us improve the clinical outcome of radiotherapy. Thus, gene signatures have been demonstrated to be specific to tumor types; hence, cell dependency must be considered in future treatment planning. Molecular and clinical features affect the results of radiotherapy. Thus, using gene technology and molecular information, it is possible to improve therapy and reduce the side effects of therapeutic radiation use. Knowing the different genes involved in tumorigenesis will make possible the improvement of clinical chemotherapy. Thus, gene signatures of the growth factors involved in different tumor types should be considered in future treatment planning, developing an original approach never considered before for any type of cancer.

## Figures and Tables

**Table 1:** Gene expression and the clinical stage factors across various breast cancer subtypes.

Breast Cancer	*FGF2*	*FGFBP1*	*TGFA*	*TGFBR3*	*IGF1R*
BRCA (*n* = 1100)	3, 4 ***	3, 4 ***	3, 4 ***	3, 4 ***	3, 4 ***
BRCA-Basal (*n* = 191)	N.S.	N.S.	N.S.	N.S.	N.S.
BRCA-Her2 (*n* = 82)	4 *	4 *	4 *	4 **	4 *
BRCA-LumA (*n* = 568)	4 ***	4 **	4 ***	4 ***	4 ***
BRCA-LumB (*n* = 219)	4 **	4 *	4 **	4 **	4 *

The statistical significance is annotated by the number of asterisks (*: *p*-value < 0.05, **: *p*-value < 0.01, and ***: *p*-value < 0.001); 3, 4: clinical stage factor; N.S.: not significant.

## Data Availability

TIMER2.0 is freely available at http://timer.cistrome.org (accessed on 6 August 2021). UCSC Xena online exploration tools are freely available at http://xena.ucsc.edu/ (accessed on 20 August 2021).
